# Incidence of hospital referred head injuries in Norway: A population based survey from the Stavanger region

**DOI:** 10.1186/1757-7241-17-6

**Published:** 2009-02-20

**Authors:** Ben Heskestad, Roald Baardsen, Eirik Helseth, Bertil Romner, Knut Waterloo, Tor Ingebrigtsen

**Affiliations:** 1 Department of Neurosurgery, Ullevål University Hospital, Oslo, Norway; 2 Department of Neurosurgery, Stavanger University Hospital, Stavanger, Norway; 3 Department of Neurosurgery, Institute for Clinical Medicine, University of Tromsø, Tromsø, Norway; 4 Department of neurology, University Hospital of North Norway, Tromsø, Norway

## Abstract

**Background:**

In three previous Norwegian studies conducted between 1974 and 1993, the annual incidence rates of hospital admitted head injuries were 236, 200 and 169 per 100,000 population. The aim of this study was to describe the incidence of head injury in the Stavanger region and to compare it with previous Norwegian studies.

**Methods:**

All head injured patients referred to Stavanger University Hospital during a one-year period (2003) were registered in a partly prospective and partly retrospective study. The catchment area for the hospital is strictly defined to a local population of 283,317 inhabitants (2003).

**Results:**

The annual incidence rate was 207/100,000 population for hospital referred head injury and 157/100,000 population for hospital admitted head injury. High age- and sex specific incidence rates were observed among the oldest, and the highest rate (882/100,000) among men above 90 years. More than 50% of the injuries were caused by falls.

**Conclusion:**

Comparison with previous Norwegian studies indicates decreasing annual incidence rates for hospital admitted head injury during the last 30 years.

## Introduction

Head injury accounts for the majority of trauma deaths and contributes strongly to costs in the health care system [1,2]. Epidemiological studies provide information about the occurrence of head injury, the nature of its distribution, the severity of cases and causes, and is therefore required for prevention and effective treatment for head injured patients.

Reviews of head injury epidemiology conclude that comparison of incidence rates from different studies is difficult because of variations in definitions and inclusion criteria, admission policies and health care systems within and between countries [1,2]. The number of patients presented to a hospital after head trauma is most likely the best measurement for the incidence of head injury [2]. In a recent review by Tagliaferri et al. [1], annual incidence rates of hospital admitted head injuries varied between 91 and 546 per 100,000 population per year in European countries. The observed incidence rates have typically been high in South Europe and low in Scandinavia. In three previous Norwegian studies conducted in 1974, 1979 and 1993, the annual incidence rates were 236, 200 and 169 per 100,000 population, respectively [3-5]. A Swedish study with data collection from 1987–2000 including 22,000 patients showed an average annual incidence rate of 229 per 100,000 population [6], while a recent study with data collection in 2005 and 2006 from the Norwegian capital region showed a surprisingly low annual incidence rate of 83.3 per 100,000 population [7].

The aim of the present study was to describe the incidence of head injury in the Stavanger region and to compare it with previous Norwegian studies.

## Materials and methods

### Study region

The University Hospital of Stavanger is located in the south-western part of Norway as a university hospital for a population of 385,020 inhabitants. It also serves as a local hospital for 283,317 of these inhabitants (Statistics of Norway, 2003). The catchment area for the local hospital function is strictly defined to the city of Stavanger and 15 surrounding municipalities. There are no other hospitals or private clinics in this region.

### Study population

In Norway, the primary health care system operates emergency services staffed with general practitioners (GP), while the secondary health care system operates hospitals. Most head injured patients are seen by a GP at a primary health care facility before eventual referral to hospital, but severely injured patients are transported directly to hospital by ambulance, and low numbers of patients not complying with the system may access hospitals directly.

This population based survey includes all head-injured patients (n = 585) referred to any department at the hospital during a one-year period (2003). Patients referred to the emergency room but discharged after assessment were also included.

Data collection was prospective for patients 15 years and older (n = 403), and retrospective for patients younger than 15 years (n = 182). Case ascertainment procedures in the prospective part of the study included continuous data collection by the physician on call in all cases, by completion of a registration form with demographic and clinical data. Electronic searches in the hospitals patient administrative database was performed every second day for identification of eventual patients not included, and the same data collection form was completed by the primary author within three days after injury by review of the patient files. The electronic search identified all patients with ICD-10 codes S00 through S09 with subgroups.

Patients younger than 15 years were identified retrospectively with a similar electronic search, and the data collection form was completed by the primary author by review of patients files.

### Inclusion and exclusion criteria

For the purpose of this study, head injury was defined as physical damage to the brain or skull caused by external force.

Patients with isolated injuries to the scalp, face or cervical spine and patients with birth injuries were excluded. Head injured patients referred to our hospital who were not registered in the Norwegian Population Registry as citizens in the hospitals catchment area were not included in the study. Incidence rates were calculated on the basis of demographic data from 2003 (283,317 inhabitants; source: Norwegian Social Science Data Services).

Prior to the data collection period the Scandinavian guidelines for management of minimal, mild and moderate head injuries had been implemented as a standard for assessment and treatment of head injured patients in our hospital [8]. This guideline classifies patients according to the Head Injury Severity Scale, which is based on Glasgow Coma Scale (GCS) scoring, the presence (and duration) or absence of loss of consciousness in the history, and the presence or absence of focal neurological deficits [9]. This scale was developed after examination of the outcome of almost 25,000 head-injured patients. It divides head injury into four severity grades: minimal, mild, moderate and severe. We applied the Scandinavian guidelines because they successfully separate minimal injuries with negligible risk of complications from mild and severer injuries requiring computerised tomography or observation [8,10].

### Statistics

We used the Statistical Package for the Social Sciences (SPSS) for Windows (release 14.0; SPSS Inc., Chicago, IL) for all statistical analyses. Comparisons of multiple proportions were performed with the Kruskal-Wallis H test. P-values < 0.05 were considered statistically significant.

## Results

### Incidence rates and severity distribution

During the year 2003, 585 head injured patients were referred to our hospital and included in this study. The annual incidence rate was 207/100,000 population. After evaluation in the emergency-room, 446 (76%) were admitted for hospitalisation giving an admission rate of 157/100,000 population. The distribution of head injury severity according to the HISS at the time of examination was 153 minimal, 339 mild, 16 moderate and 77 severe. The admission rate increased significantly (p < 0.001) with severity of injury (73% for minimal, 88% for mild, 100% for moderate and 97% for severe).

**Figure 1:**
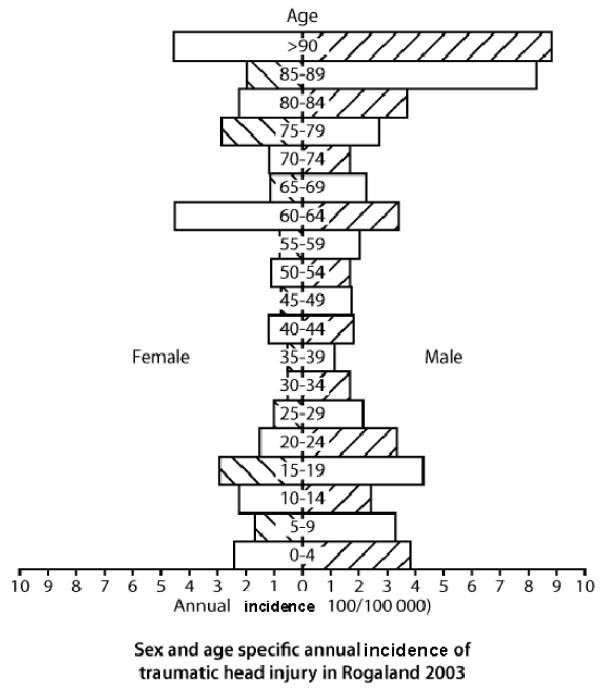
**Population tree depicting age- and sex-specific annual incidence rates (cases/100,000 population) of hospital-referred head injury in the Stavanger region**.

The sex-specific incidence rates were 258/100,000 for males and 156/100,000 for females, giving a male preponderance of 1.7:1.0. High age-specific incidence rates for men were found in the age groups 10–24 years, with the peak (428/100,000) among teenagers between 15 and 19 years (Fig. 1.) The same trend was observed for women, with a less pronounced overrepresentation of teenagers. In both genders, the highest age and sex specific incidence rates were observed among the oldest, with the highest rate in the study (882/100,000) for men above 90 years.

### Cause of injury

The injuries were caused by falls in 299 (51%), traffic accidents in 126 (21%), assaults in 81 (14%) and other causes in 79 (14%) cases. The male: female ratio was highest for head injury caused by assaults (2.9:1) and lowest for head injury caused by traffic accidents (1.4:1).

**Figure 2:**
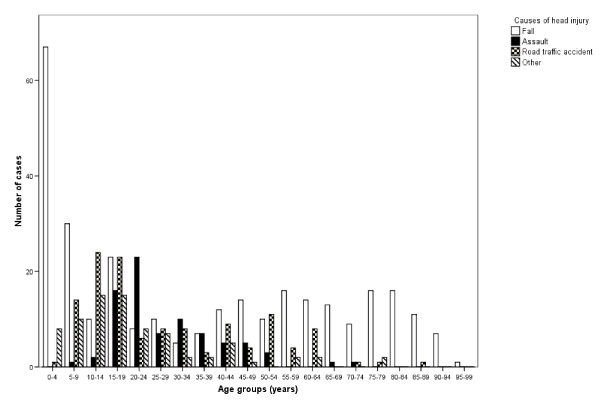
**Causes of head injury by specific age groups**.

Falls were the most common cause of injury in children younger than 10 years and in adults 40 years and older as shown in Fig. 2. In the age groups between 10 and 40 years, falls and road traffic accidents accounted for approximately equal proportions of the injuries, while assaults were observed mainly between 15 and 24 years. We did not observe any significant relation between the cause of head injury and head injury severity.

### Radiological Findings

Table 1 shows that the use of cranial computerised tomography (CT) increased significantly (p < 0.001) with severity of injury, from 55% in minimal injuries to 99% in severe injuries. In those examined with CT, a skull fracture or intracranial lesion was revealed in 1% of minimal, 4% of mild, 56% of moderate and 99% of severe head injuries. A total number of 12 (2%) epidural hematomas were observed in the 585 patients, and 11 of these occurred in patients with injuries classified as moderate or severe.

**Table 1:** Distribution of head injury cases, use of computed tomography (CT) and CT findings in 585 head injured patients.

				Injuries revealed by CT (n = number of observations)				
				
Severity category	Number of cases	Proportion examined with CT (n = number of cases)	Proportion with CT verified injury (n = number of cases)	Cerebral contusion	tSAH	SDH	EDH	Fracture
Minimal	153	84 (55%)	1 (1%)	0	0	0	0	1
Mild	339	284 (84%)	15 (4%)	3	1	2	1	8
Moderate	16	16 (100%)	9 (56%)	3	4	2	1	5
Severe	77	77 (100%)	76 (99%)	38	23	35	10	43

Total	585	461 (79%)	101 (22%)	44	28	39	12	57

tSAH: traumatic subarachnoid haemorrhage; SDH: subdural haematoma; EDH: epidural haematoma

## Discussion

The main finding in this study is an annual incidence rate of 207/100,000 population for hospital referred head injury and 157/100,000 population for hospital admitted head injury. High age- and sex specific incidence rates were observed among the oldest and the highest rate (882/100,000) for men above 90 years. More than 50% of the injuries were caused by falls.

A recent review of European head injury epidemiology included 23 studies from 14 countries and concluded that an overall incidence rate of about 235/100,000 population per year could be derived [1]. Comparison of head injury incidence rates from different studies is difficult due to variability in inclusion criteria, admission policies and health care systems. Caution must therefore be taken when interpreting these results from the present study. Comparison with previous Norwegian and Nordic studies is probably most relevant.

Incidence rates from three previous Norwegian studies conducted in 1974, 1979 and 1993 are available for hospital admissions only [3-5]. Interestingly, a clear trend towards reduced incidence is present, as the annual admission rates in these studies were 200, 236 and 169/100,000 population respectively. In the present study, a further reduction to 157/100,000 population was observed. A recent study from the Norwegian capital region (Oslo) reported a surprisingly low incidence of hospital admitted patients (83.3/100,000 population) [7]. The authors speculate that this might be the result from effective prevention programs, but they may not have taken into consideration that a significant proportion of head injured patients in Oslo is treated at an advanced outpatient clinic with facilities for overnight observation.

A national Danish study for the period 1979–1996 reported a decrease in the incidence of hospital admitted head injury from 265 to 157 per 100,000 population [11]. This is almost identical with the trend in Norway. In Finland, a study based on data from the National Hospital Discharge Register for the period 1991 to 2005 showed a stable incidence [12]. Taken together, the incidence of hospital admitted head injuries in the Nordic countries seems to have been significantly reduced during the last 30 years, and especially the 1980's and 1990's.

Most studies report an overrepresentation of teenagers and young adults, especially men [2,5]. Comparison between our previous study in Tromsø, Norway, in 1993 and the present survey indicates a modification of this, as the age- and sex specific rates for male teenagers in the previous study was about 500/100,000 population per year, while the corresponding figure in Stavanger in 2003 was approximately 300/100,000 per year. It could be speculated that preventive measures such as bike helmets and other efforts to reduce the frequency of traffic accidents have been specifically effective in these age groups.

The very high incidence rate for head injuries caused by falls among old people above 80 years is an interesting observation in the present study, as the incidence for those above 80 is twice as high as for teenagers. A trend towards reduced incidence rates among teenagers and young adults and increased rates for the elderly is consistent in other Nordic studies [5-7,11]. This could be an effect of extensive injury preventive measures directed towards traffic accidents and sports injuries [13]. Now, increased focus on prevention of falls among the elderly may be more efficient in achieving further reduction in head injury incidence.

The Scandinavian guidelines for management of minimal, mild and moderate head injuries recommend outpatient management of patients with head injuries classified as minimal or mild [8]. Implementation of this practice has probably contributed to the reduction of hospital admission for head injuries [14]. On the other hand the high admission rate (73%) and the frequent use of CT (55%) for patients with injuries classified as minimal in the present study, is surprising and not in accordance with the management recommended by the guidelines published by the Scandinavian Neurotrauma Committee [8]. This practice contributes to a high admission rate and could influence comparison with other studies. A separate study of guideline compliance would be reasonable.

This study has limitations. The data collection was partially retrospective, which may have influenced the accuracy of the inclusion procedure and the classification of the injuries. The study sample is relatively small and this implies a risk for random variation (type II error).

## Conclusion

Comparison with previous Norwegian studies indicates decreasing annual incidence rates for traumatic head injury during the last 30 years.

## Competing interests

The authors declare that they have no competing interests.

## Authors' contributions

BH and RB designed the study. BH and RB conducted data collection. All authors participated in data interpretation, literature research and preparation of the manuscript.
